# Supplementary material for: An advanced cell cycle tag toolbox reveals principles underlying temporal control of structure-selective nucleases
Source: eLife. 2020 May 1;9:e52459. doi: 10.7554/eLife.52459 (PMC7220381; doi:10.7554/eLife.52459)
Supplement: Supplementary file 1. — Table 1. Yeast strains used in this study. Table 2. Cell cycle tagging plasmids used in this study Table 3. Other plasmids used in this study [file elife-52459-supp1.docx]

**Supplementary file 1**

Table 1: Yeast strains used in this study

| Strain | Relevant genotype | Source |
| --- | --- | --- |
| W303a | MATa ade2-1 ura3-1 his3-11,15 trp1-1 leu2-3,112 can1-100 | (Rothstein, 1983) |
| yJB825 | W303a  his3::natNT2-Clb5 promoter-Clb5 N-terminus-3FLAG-yeGFP::HIS3 | This study |
| yJB878 | W303a  his3::natNT2-Clb5 promoter (-20bp)-Clb5 N-terminus-3FLAG-yeGFP::HIS3 | This study |
| yJB879 | W303a  his3::natNT2- Clb5 promoter (-40bp)-Clb5 N-terminus-3FLAG-yeGFP::HIS3 | This study |
| yJB880 | W303a  his3::natNT2-Clb5 promoter 3 (-60bp) -Clb5 N-terminus -3FLAG-yeGFP::HIS3 | This study |
| yJB881 | W303a  his3::natNT2- Clb5 promoter (-80bp)-Clb5 N-terminus-3FLAG-yeGFP::HIS3 | This study |
| yJB827 | W303a  his3::natNT2-Clb6 promoter-Clb6 N-terminus-3FLAG-yeGFP:: HIS3 | This study |
| yJB894 | W303a  his3::natNT2-Clb6 promoter (-20bp)-Clb6 N-terminus-3FLAG-yeGFP::HIS3 | This study |
| yJB895 | W303a  his3::natNT2-Clb6 promoter (-40bp)-Clb6 N-terminus-3FLAG-yeGFP::HIS3 | This study |
| yJB896 | W303a  his3::natNT2-Clb6 promoter (-60bp)-Clb6 N-terminus-3FLAG-yeGFP::HIS3 | This study |
| yJB897 | W303a  his3::natNT2-Clb6 promoter (-80bp)-Clb6 N-terminus-3FLAG-yeGFP::HIS3 | This study |
| yJB828 | W303a  his3::natNT2-Clb1 promoter-Clb1 N-terminus-3FLAG-yeGFP::HIS3 | This study |
| yJB855 | W303a  his3::natNT2-Clb1 promoter (-20bp) -Clb1 N-terminus -3FLAG-yeGFP::HIS3 | This study |
| yJB856 | W303a  his3::natNT2-Clb1 promoter (-40bp) -Clb1 N-terminus -3FLAG-yeGFP::HIS3 | This study |
| yJB857 | W303a  his3::natNT2-Clb1 promoter (-60bp) -Clb1 N-terminus -3FLAG-yeGFP::::HIS3 | This study |
| yJB858 | W303a  his3::natNT2-Clb1 promoter (-80bp) -Clb1 N-terminus -3FLAG-yeGFP::::HIS3 | This study |
| yJB859 | W303a  his3::natNT2-Clb1 promoter (-100bp) -Clb1 N-terminus -3FLAG-yeGFP::::HIS3 | This study |
| yJB860 | W303a  his3::natNT2-Clb1 promoter (-150bp) -Clb1 N-terminus -3FLAG-yeGFP::::HIS3 | This study |
| yJB861 | W303a  his3::natNT2-Clb1 promoter (-200bp) -Clb1 N-terminus -3FLAG-yeGFP::::HIS3 | This study |
| yJB826 | W303a  his3::natNT2-Clb2 promoter-Clb2 N-terminus-3FLAG-yeGFP::::HIS3 | This study |
| yJB848 | W303a  his3::natNT2-Clb2 promoter (-20bp)-Clb2 N-terminus-3FLAG-yeGFP::::HIS3 | This study |
| yJB849 | W303a  his3::natNT2-Clb2 promoter (-40bp) -Clb2 N-terminus -3FLAG-yeGFP::HIS3 | This study |
| yJB850 | W303a  his3::natNT2-Clb2 promoter (-60bp) -Clb2 N-terminus -3FLAG-yeGFP::HIS3 | This study |
| yJB851 | W303a  his3::natNT2-Clb2 promoter (-80bp) -Clb2 N-terminus -3FLAG-yeGFP::HIS3 | This study |
| yJB852 | W303a  his3::natNT2-Clb2 promoter (-100bp) -Clb2 N-terminus -3FLAG-yeGFP::HIS3 | This study |
| yJB853 | W303a  his3::natNT2-Clb2 promoter (-150bp) -Clb2 N-terminus -3FLAG-yeGFP::HIS3 | This study |
| yJB854 | W303a  his3::natNT2-Clb2 promoter (-200bp) -Clb2 N-terminus -3FLAG-yeGFP::HIS3 | This study |
| yJB892 | W303a  his3::natNT2- Clb1 promoter-Clb2 N-terminus-3FLAG-yeGFP::HIS3 | This study |
| yJB997 | W303a  his3::natNT2- Clb1 promoter (-150bp)-Clb2 N-terminus-3FLAG-yeGFP::HIS3 | This study |
| yJB893 | W303a  his3::natNT2- Clb5 promoter-Clb6 N-terminus-3FLAG-yeGFP::HIS3 | This study |
| yJB870 | W303a  his3::natNT2-Sic1 promoter-Sic1 N-terminus-3FLAG-yeGFP::HIS3 | This study |
| yJB871 | W303a  his3::natNT2- Sic1 promoter (-20bp)-Sic1 N-terminus-3FLAG-yeGFP::HIS3 | This study |
| yJB872 | W303a  his3::natNT2- Sic1 promoter (-40bp) -Sic1 N-terminus -3FLAG-yeGFP::HIS3 | This study |
| yJB1000 | W303a  his3::natNT2- Sic1 promoter (-22bp u(ATA)ATG) -Sic1 N-terminus -3FLAG-yeGFP::HIS3 | This study |
| yJB1001 | W303a  his3::natNT2- Sic1 promoter (-22bp u(GTA)ATG) -Sic1 N-terminus -3FLAG-yeGFP::HIS3 | This study |
| yJB1025 | W303a  his3::natNT2- Sic1 promoter (-22bp u(CTA)ATG) -Sic1 N-terminus -3FLAG-yeGFP::HIS3 | This study |
| yJB1026 | W303a  his3::natNT2- Sic1 promoter (-22bp u(TTA)ATG) -Sic1 N-terminus -3FLAG-yeGFP::HIS3 | This study |
| yJB838 | W303a  TRP1-Clb6 promoter-Clb6 N-terminus-9MYC-Xrs2 | This study |
| yJB839 | W303a  TRP1-Clb1 promoter-Clb2 N-terminus-9MYC-Xrs2 | This study |
| yJB886 | W303a  Xrs2-9MYC-hphNT1 | This study |
| yJB891 | W303a  NatNT2-Clb6 promoter-Clb6 N-terminus-3FLAG-Rad52 | This study |
| yJB809 | W303a  natNT2-Clb1 promoter-Clb2N-terminus-3FLAG-Rad52 | This study |
| yJB808 | W303a  Rad52-3FLAG-natNT2 | This study |
| yJB833 | W303a  natNT2-Clb6 promoter-Clb6 N-terminus-3FLAG-Fun30 | This study |
| yJB801 | W303a  natNT2-Clb1 promoter-Clb2 N-terminus-3FLAG-Fun30 | This study |
| ySB758 | W303  Fun30∆::kanMX4 | (Bantele et al., 2017) |
| yJB888 | W303a  natNT2-Clb6 promoter-Clb6 N-terminus-3FLAG-Sgs1 | This study |
| yJB797 | W303a  natNT2-Clb1 promoter-Clb2 N-terminus-3FLAG-Sgs1 | This study |
| yJB613 | W303a  Sgs1-3FLAG-hphNT1 | This study |
| yJB323 | W303a  Mms4-3FLAG-hphNT1, Mus81-9MYC-hphNT1 | This study |
| yJB489 | W303a  natNT2-Clb1 promotor-Clb2 N-terminus-3FLAG -Mms4, TRP1-Clb1 promotor-Clb2 N-terminus-9MYC-Mus81 | This study |
| yJB490 | W303a  natNT2-Clb5 promoter-Clb6 N-terminus-3FLAG -Mms4, TRP1-Clb5 promoter-Clb6 N-terminus-9MYC-Mus81 | This study |
| yMC9 | W303a  mus81∆::natNT2 | This study |
| yDG208 | W303a  RAD5+ | (Princz et al., 2017) |
| yJB670 | W303 alpha  RAD5+, mus81∆::natNT2 | This study |
| yDG335 | W303 a  RAD5+, mus81∆::hphNT1 | This study |
| yJB517 | W303a  Rad5+, natNT2-Clb1 promoter-Clb2 N-terminus-3FLAG -Mms4, TRP1-Clb1 promoter-Clb2 N-terminus-9MYC-Mus81 | This study |
| yJB519 | W303a  Rad5+, natNT2-Clb5 promoter-Clb6 N-terminus-3FLAG -Mms4, TRP1-Clb5 promoter-Clb6 N-terminus-9MYC-Mus81 | This study |
| yJB941 | W303a  Rad5+, natNT2-Clb6 promoter (-80bp)-Clb6 N-terminus-3FLAG-Mms4, TRP1-Clb6 promoter (-80bp)-Clb6 N-terminus-9MYC-Mus81 | This study |
| yJB918 | W303a  Rad5+, TRP1-Clb1 promoter (-150bp)-Clb2 N-terminus-9MYC-Mus81, natNT2-Clb1 promoter (-150bp)-Clb2 N-terminus-3FLAG-Mms4 | This study |
| yJB1078 | W303a  Rad5+, mms4∆::natNT2, Ura3::Clb5 promoter-Clb6 N-terminus-3FLAG -mms4 14A::URA3, TRP1-Clb5 promoter-Clb6 N-terminus-9MYC-Mus81 | This study |
| yJB1057 | W303a  Rad5+, TRP1-Clb1 promoter (-150bp)-Clb2 N-terminus-9MYC-Mus81, natNT2-Clb1 promoter (-150bp)-Clb2 N-terminus-3FLAG-Mms4, leu2::Clb6 promoter (-80bp)-Clb6 N-terminus-9MYC-Mus81:.LEU2, ura3::Clb6 promoter (-80bp)-Clb6 N-terminus-3FLAG-Mms4::URA3 | This study |
| yJB694 | W303a  rnh1∆::HIS3, rnh201∆::LEU2 | This study |
| yJB638 | W303a  mus81∆::kanMX6, rnh1∆::HIS3, rnh201∆::LEU2 | This study |
| yJB639 | W303a  natNT2-Clb1 promotor-Clb2 N-terminus-3FLAG-Mms4, TRP1-Clb1 promotor-Clb2 N-terminus-9MYC-Mus81, rnh1∆::HIS3, rnh201∆::LEU2 | This study |
| yJB641 | W303a  natNT2-Clb5 promoter-Clb6 N-terminus-3FLAG-Mms4, TRP1-Clb5 promoter-Clb6 N-terminus-9MYC-Mus81, rnh1∆::HIS3, rnh201∆::LEU2 | This study |
| yJB920 | W303 a  natNT2-Clb1 promoter (-150bp)-Clb2 N-terminus-3FLAG-Mms4, TRP1-Clb1 promoter (-150bp)-Clb2 N-terminus-9MYC-Mus81, rnh1∆::HIS3, rnh201∆::LEU2 | This study |
| yJB953 | W303a  natNT2-Clb6 promoter (-80bp)-Clb6 N-terminus-3FLAG-Mms4, TRP1- Clb6 promoter (-80bp)-Clb6 N-terminus-9MYC-Mus81, rnh1∆::HIS3, rnh201∆::LEU2 | This study |
| yJB721 | W303a  Cir0, leu2::pGal10-FLP(H305L)::LEU2, kanMX4-FRT-ChrIV | This study |
| yJB727 | W303a  Cir0, leu2::pGal10-FLP(H305L)::LEU2, kanMX4-FRT -ChrIV, yen1∆::hphNT1 | This study |
| yJB745 | W303a  Cir0, leu2::pGal10-FLP(H305L)::LEU2, kanMX4-FRT-ChrIV, yen1∆::hphNT1, mus81∆::natNT2 | This study |
| yJB746 | W303a  Cir0, leu2::pGal10-FLP(H305L)::LEU2, kanMX4-FRT-ChrIV, yen1∆::hphNT1, TRP1-Clb1 promoter-Clb2 N-terminus-9MYC-Mus81, natNT2-Clb1 promoter-Clb2 N-terminus-3FLAG-Mms4 | This study |
| yJB747 | W303a  Cir0, leu2::pGal10-FLP(H305L)::LEU2, kanMX4-FRT-ChrIV, yen1∆::hphNT1, TRP1-Clb5 promoter-Clb6 N-terminus-9MYC-Mus81, natNT2-Clb5 promoter-Clb6 N-terminus-3FLAG-Mms4 | This study |
| yJB724 | W303a  Cir0, leu2::pGal10-FLP(H305L)::LEU2, kanMX4-FRT-ChrIV, rad52∆::HIS3 | This study |
| yJB723 | W303a  Cir0, leu2::pGal10-FLP(H305L)::LEU2, kanMX4-FRT-ChrVI | This study |
| yJB728 | W303a  Cir0, leu2::pGal10-FLP(H305L)::LEU2, kanMX4-FRT-ChrVI, yen1∆::hphNT1 | This study |
| yJB742 | W303a  Cir0, leu2::pGal10-FLP(H305L)::LEU2, kanMX4-FRT-ChrVI, yen1∆::hphNT1, mus81∆::natNT2 | This study |
| yJB743 | W303a  Cir0, leu2::pGal10-FLP(H305L)::LEU2, kanMX4-FRT-ChrVI, yen1∆::hphNT1, TRP1-Clb1 promoter-Clb2 N-terminus-9MYC-Mus81, natNT2-Clb1 promoter-Clb2 N-terminus-3FLAG-Mms4 | This study |
| yJB744 | W303a  Cir0, leu2::pGal10-FLP(H305L)::LEU2, kanMX4-FRT-ChrVI, yen1∆::hphNT1, TRP1-Clb5 promoter-Clb6 N-terminus-9MYC-Mus81, natNT2-Clb5 promoter-Clb6 N-terminus-3FLAG-Mms4 | This study |
| yJB725 | W303a  Cir0, leu2::pGal10-FLP(H305L)::LEU2, kanMX4-FRT-ChrVI, rad52∆::HIS3 | This study |
| yJB967 | W303a  Cir0, leu2::pGal10-FLP(H305L)::LEU2, kanMX4-FRT-ChrIV, yen1∆::hphNT1, TRP1- Clb6 promoter (-80bp)-Clb6 N-terminus-9MYC-Mus81, natNT2- Clb6 promoter –(80bp)-Clb6 N-terminus-3FLAG-Mms4 | This study |
| yJB829 | W303a  Cir0, leu2::pGal10-FLP(H305L)::LEU2, kanMX4-FRT-ChrIV, yen1∆::hphNT1, natNT2-Clb1 promoter (-150bp)-Clb2 N-terminus-3FLAG-Mms4, TRP1-Clb1 promoter (-150bp)-Clb2 N-terminus-9MYC-Mus81 | This study |
| yJB968 | W303a  Cir0, leu2::pGal10-FLP(H305L)::LEU2, kanMX4-FRT -ChrVI,yen1∆::hphNT1, TRP1-Clb6 promoter (-80bp)-Clb6 N-terminus-9MYC-Mus81, natNT2- Clb6 promoter (-80bp)-Clb6 N-terminus-3FLAG-Mms4 | This study |
| yJB831 | W303a  Cir0, leu2::pGal10-FLP(H305L)::LEU2, kanMX4-FRT-ChrVI, yen1∆::hphNT1, natNT2-Clb1 promoter (-150bp)-Clb2 N-terminus-3FLAG-Mms4, TRP1-Clb1 promoter (-150bp)-Clb2 N-terminus-9MYC-Mus81 | This study |
| yJB931 | W303a  natNT2-Clb6 promoter (-80bp)-Clb6 N-terminus-3FLAG-Mms4, TRP1-Clb6 promoter (-80bp)-Clb6 N-terminus-9MYC-Mus81 | This study |
| yJB764 | W303a  natNT2-Clb1 promoter (-150bp)-Clb2 N-terminus-3FLAG-Mms4, TRP1-Clb1 promoter (-150bp)-Clb2 N-terminus-9MYC-Mus81 | This study |
| yJB197 | W303alpha  sgs1∆::natNT2 | This study |
| yJB535 | W303diploid  MATa/MATalpha, ade2-n/ade2-I, his3∆::natMX4/his3∆::hphMX4, met22∆::kIURA3/MET22, LYS2/lys2∆::pGAL-IsceI | Princz et al., 2017 |
| yJB607 | W303diploid  MATa/MATalpha, ade2-n/ade2-I, his3∆::natMX4/his3∆::hphMX4, met22∆::kIURA3/MET22, LYS2/ lys2::GAL-ISCEI, mus81∆::kanMX6/mus81∆::kanMX6, mms4∆::TRP1/mms4∆::TRP11 | Princz et al., 2017 |
| yJB668 | W303diploid  MATa/MATalpha, ade2-n/ade2-I, his3∆::natMX4/his3∆::hphMX4, met22∆::kIURA3/MET22, LYS2/ lys2::GAL-ISCEI, TRP1-Clb2 promoter-Clb1 N-terminus-9MYC-Mus81/TRP1-Clb2 promoter-Clb1 N-terminus-9MYC-Mus81, kanMX6-Clb2 promoter-Clb1 N-terminus-3FLAG-Mms4/kanMX6-Clb2 promoter-Clb1 N-terminus-3FLAG-Mms4 | This study |
| yJB669 | W303diploid  MATa/MATalpha, ade2-n/ade2-I, his3∆::natMX4/his3∆::hphMX4, met22∆::kIURA3/MET22, LYS2/ lys2::GAL-ISCEI, TRP1-Clb5 promoter-Clb6 N-terminus-9MYC-Mus81/TRP1-Clb5 promoter-Clb6 N-terminus-9MYC-Mus81, kanMX6-Clb5 promoter-Clb6 N-terminus-3FLAG-Mms4/kanMX6-Clb5 promoter-Clb6 N-terminus-3FLAG-Mms4 | This study |
| yJB1030 | W303diploid  MATa/MATalpha, ade2-n/ade2-I, his3∆::natMX4/his3∆::hphMX4, met22∆::kIURA3/MET22, LYS2/ lys2::GAL-ISCEI, TRP1-Clb1 promoter (-150bp)-Clb2 N-terminus-9MYC-Mus81/ TRP1-Clb1 promoter (-150bp)-Clb2 N-terminus-9MYC-Mus81, kanMX6-Clb1 promoter (-150bp)-Clb2 N-terminus-3FLAG-Mms4/natNT2-Clb1 promoter (-150bp)-Clb2 N-terminus-3FLAG-Mms4 | This study |
| yJB1031 | W303diploid  MATa/MATalpha, ade2-n/ade2-I, his3∆::natMX4/his3∆::hphMX4, met22∆::kIURA3/MET22, LYS2/ lys2::GAL-ISCEI, TRP1-Clb6 promoter (-80bp)-Clb6 N-terminus-9MYC-Mus81/TRP1-Clb6 promoter (-80bp)-Clb6 N-terminus-9MYC-Mus81, kanMX6-Clb6 promoter (-80bp)-Clb6 N-terminus-3FLAG-Mms4/natNT2-Clb6 promoter (-80bp)-Clb6 N-terminus-3FLAG-Mms4 | This study |
| yJB548 | W303a  RAD5+, yen1∆::kanMX6, his3::Yen1-ON-9MYC::HIS3 | This study |
| yJB561 | W303a  RAD5+, yen1∆::kanMX6, his3::Clb6 promoter-Yen1-ON-Clb6 N-terminus-9MYC::HIS3 | This study |
| yJB553 | W303a  RAD5+, yen1∆::kanMX6, his3::Clb1 promoter-Yen1-ON-Clb2 N-terminus-9MYC::HIS3 | This study |
| yJB779 | W303a  RAD5+, yen1∆::kanMX4, his3:: Sic1 promoter (-20bp)-Yen1-ON-Sic1 N-terminus-9MYC::HIS3 | This study |
| yJB775 | W303a  RAD5+, yen1∆::kanMX4, his3::Clb6 promoter (-80bp)-Yen1-ON-Clb6 N-terminus-9MYC)::HIS3 | This study |
| yJB702 | W303a  RAD5+, yen1∆:: kanMX6, his3::Clb2 promoter (-60bp)-Yen1-ON-Clb2 N-terminus-9MYC::HIS3 | This study |
| yJB1066 | W303a  RAD5+, yen1∆::kanMX4, his3:: Sic1 promoter (-22bp u(ATA)ATG)-Yen1-ON-Sic1 N-terminus-9MYC::HIS3 | This study |
| yJB654 | W303a  RAD5+, yen1∆::kanMX6, his3::Yen1-ON-9MYC::HIS3, mus81∆::natNT2 | This study |
| yJB659 | W303a  Rad5+, yen1∆::kanMX6, his3::Clb6 promoter-Yen1-ON-Clb6 N-terminus-9MYC::HIS3, mus81∆::natNT2 | This study |
| yJB657 | W303a  Rad5+, yen1∆::kanMX6, his3::Clb1 promoter-Yen1-ON-Clb2 N-terminus-9MYC::HIS3, mus81∆::natNT2 | This study |
| yJB936 | W303a  Rad5+, yen1∆::kanMX6, his3::Sic1 promoter (-20bp)-Yen1-ON-Sic1 N-terminus-9MYC::HIS3, mus81∆::natNT2 | This study |
| yJB868 | W303a  RAD5+, yen1∆::kanMX6, his3::Clb6 promoter (-80bp)-Yen1-ON-Clb6 N-terminus-9MYC::HIS3, mus81∆::natNT2 | This study |
| yJB757 | W303a  RAD5+, yen1∆:: kanMX6, his3::Clb2 promoter (-60bp)-Yen1-ON-Clb2 N-terminus-9MYC::HIS3, mus81∆::natNT2 | This study |
| yJB957 | W303a  pep4∆::LEU2, his3::Clb6-9MYC-Mus81-Gal1-10-Clb6-3FLAG-Mms4::HIS3 | This study |
| yJB958 | W303a  pep4∆::LEU2, his::Clb2-9MYC-Mus81-Gal1-10-Clb2-3FLAG-Mms4::HIS3 | This study |
| yLG47 | W303a  Pep4∆::LEU2, his::Mus81-9MYC-Gal1-10-Mms4-3FLAG::HIS3 | This study |

Table 2: Cell cycle tagging plasmids used in this study

| **cell cycle tag (CCT)** | **plasmid number** | **description** |
| --- | --- | --- |
| CCT_1 | pJB138 | pYM-N31 Clb1 promoter-Clb1 N-terminus-3FLAG |
| CCT_2 | pJB259 | pYM-N31 Clb1 promoter (-20 bp)-Clb1 N-terminus-3FLAG |
| CCT_3 | pJB260 | pYM-N31 Clb1 promoter (-40 bp)-Clb1 N-terminus-3FLAG |
| CCT_4 | pJB261 | pYM-N31 Clb1 promoter (-60 bp)-Clb1 N-terminus-3FLAG |
| CCT_5 | pJB262 | pYM-N31 Clb1 promoter (-80 bp)-Clb1 N-terminus-3FLAG |
| CCT_6 | pJB263 | pYM-N31 Clb1 promoter (-150 bp)-Clb1 N-terminus-3FLAG |
| CCT_7 | pJB264 | pYM-N31 Clb1 promoter (-200 bp)-Clb1 N-terminus-3FLAG |
| CCT_8 | pJB87 | pYM-N31 Clb2 promoter-Clb2 N-terminus-3FLAG |
| CCT_9 | pJB245 | pYM-N31 Clb2 promoter (-20 bp)-Clb2 N-terminus-3FLAG |
| CCT_10 | pJB246 | pYM-N31 Clb2 promoter (-40 bp)-Clb2 N-terminus-3FLAG |
| CCT_11 | pJB247 | pYM-N31 Clb2 promoter (-60 bp)-Clb2 N-terminus-3FLAG |
| CCT_12 | pJB248 | pYM-N31 Clb2 promoter (-80 bp)-Clb2 N-terminus-3FLAG |
| CCT_13 | pJB249 | pYM-N31 Clb2 promoter (-100 bp)-Clb2 N-terminus-3FLAG |
| CCT_14 | pJB250 | pYM-N31 Clb2 promoter (-150 bp)-Clb2 N-terminus-3FLAG |
| CCT_15 | pJB251 | pYM-N31 Clb2 promoter (-200 bp)-Clb2 N-terminus-3FLAG |
| CCT_16 | pJB139 | pYM-N31 Clb1 promoter-Clb2 N-terminus-3FLAG |
| CCT_17 | yJB306 | pYM-N31 Clb1 promoter (-20 bp)-Clb2 N-terminus-3FLAG |
| CCT_18 | yJB307 | pYM-N31 Clb1 promoter (-40 bp)-Clb2 N-terminus-3FLAG |
| CCT_19 | yJB308 | pYM-N31 Clb1 promoter (-60 bp)-Clb2 N-terminus-3FLAG |
| CCT_20 | yJB309 | pYM-N31 Clb1 promoter (-80 bp)-Clb2 N-terminus-3FLAG |
| CCT_21 | yJB310 | pYM-N31 Clb1 promoter (-100 bp)-Clb2 N-terminus-3FLAG |
| CCT_22 | yJB311 | pYM-N31 Clb1 promoter (-150 bp)-Clb2 N-terminus-3FLAG |
| CCT_23 | yJB312 | pYM-N31 Clb1 promoter (-200 bp)-Clb2 N-terminus-3FLAG |
| CCT_24 | pJB83 | pYM-N31 Clb5 promoter-Clb5 N-terminus-3FLAG |
| CCT_25 | pJB238 | pYM-N31 Clb5 promoter (-20 bp)-Clb5 N-terminus-3FLAG |
| CCT_26 | pJB239 | pYM-N31 Clb5 promoter (-40 bp)-Clb5 N-terminus-3FLAG |
| CCT_27 | pJB240 | pYM-N31 Clb5 promoter (-60 bp)-Clb5 N-terminus-3FLAG |
| CCT_28 | pJB241 | pYM-N31 Clb5 promoter (-80 bp)-Clb5 N-terminus-3FLAG |
| CCT_29 | pJB122 | pYM-N31 Clb6 promoter-Clb6 N-terminus-3FLAG |
| CCT_30 | pJB299 | pYM-N31 Clb6 promoter (-20 bp)-Clb6 N-terminus-3FLAG |
| CCT_31 | pJB300 | pYM-N31 Clb6 promoter (-40 bp)-Clb6 N-terminus-3FLAG |
| CCT_32 | pJB301 | pYM-N31 Clb6 promoter (-60 bp)-Clb6 N-terminus-3FLAG |
| CCT_33 | pJB302 | pYM-N31 Clb6 promoter (-80 bp)-Clb6 N-terminus-3FLAG |
| CCT_34 | pJB140 | pYM-N31 Clb5 promoter-Clb6 N-terminus-3FLAG |
| CCT_35 | pJB252 | pYM-N31 Clb5 promoter (-20 bp)-Clb6 N-terminus-3FLAG |
| CCT_36 | pJB253 | pYM-N31 Clb5 promoter (-40 bp)-Clb6 N-terminus-3FLAG |
| CCT_38 | pJB254 | pYM-N31 Clb5 promoter (-60 bp)-Clb6 N-terminus-3FLAG |
| CCT_39 | pJB255 | pYM-N31 Clb5 promoter (-80 bp)-Clb6 N-terminus-3FLAG |
| CCT_40 | pJB237 | pYM-N31 Sic1 promoter-Sic1 N-terminus-3FLAG |
| CCT_41 | pJB238 | pYM-N31 Sic1 promoter (-20 bp)-Sic1 N-terminus-3FLAG |
| CCT_42 | pJB239 | pYM-N31 Sic1 promoter (-40 bp)-Sic1 N-terminus-3FLAG |
| CCT_43 | pJB321 | pYM-N31 Sic1 promoter (-22 bp u(ATA)ATG)-Sic1 N-terminus-3FLAG |
| CCT_44 | pJB322 | pYM-N31 Sic1 promoter (-22 bp u(GTA)ATG)-Sic1 N-terminus-3FLAG |
| CCT_45 | pJB329 | pYM-N31 Sic1 promoter (-22 bp u(CTA)ATG)-Sic1 N-terminus-3FLAG |
| CCT_46 | pJB330 | pYM-N31 Sic1 promoter (-22 bp u(TTA)ATG)-Sic1 N-terminus-3FLAG |

Table 3: Other plasmids used in this study

| **plasmid number** | **description** |
| --- | --- |
| pJB134 | pRS303 Yen1-ON-9MYC |
| pJB154 | pRS303 Clb1 promoter-Yen1-ON-Clb2 N-terminus-9MYC |
| pJB183 | pRS303 Clb2 promoter (-60 bp)-Yen1-ON-Clb2 N-terminus-9MYC |
| pJB147 | pRS303 Clb6 promoter-Yen1-ON-Clb6 N-terminus-9MYC |
| pJB215 | pRS303 Clb6 promoter (-80bp)-Yen1-ON-Clb6 N-terminus-9MYC |
| pJB219 | pRS303 Sic1 promoter (-20 bp)-Yen1-ON-Sic1 N-terminus-9MYC |
| pJB337 | pRS303 Sic1 promoter (-22 bp u(ATA)ATG)-Yen1-ON-Sic1 N-terminus-9MYC |
| pJB164 | pRS303 Mus81-9MYC Gal1-10 Mms4-3FLAG |
| pJB295 | pRS303 Clb6 N-terminus-9MYC-Mus81 Gal1-10 Clb6 N-terminus-3FLAG-Mms4 |
| pJB296 | pRS303 Clb2 N-terminus-9MYC-Mus81 Gal1-10 Clb2 N-terminus-3FLAG-Mms4 |

**Supplementary References**

Bantele, S.C., Ferreira, P., Gritenaite, D., Boos, D., and Pfander, B. (2017). Targeting of the Fun30 nucleosome remodeller by the Dpb11 scaffold facilitates cell cycle-regulated DNA end resection. Elife *6*.

Princz, L.N., Wild, P., Bittmann, J., Aguado, F.J., Blanco, M.G., Matos, J., and Pfander, B. (2017). Dbf4-dependent kinase and the Rtt107 scaffold promote Mus81-Mms4 resolvase activation during mitosis. EMBO J.

Rothstein, R.J. (1983). One-step gene disruption in yeast. Methods Enzymol *101*, 202-211.
