# Supplementary material for: An advanced cell cycle tag toolbox reveals principles underlying temporal control of structure-selective nucleases
Source: eLife. 2020 May 1;9:e52459. doi: 10.7554/eLife.52459 (PMC7220381; doi:10.7554/eLife.52459)
Supplement: Transparent reporting form [file elife-52459-transrepform.docx]

***eLife’s* transparent reporting form**

We encourage authors to provide detailed information *within their submission* to facilitate the interpretation and replication of experiments. Authors can upload supporting documentation to indicate the use of appropriate reporting guidelines for health-related research (see [EQUATOR Network](http://www.equator-network.org/%20)), life science research (see the [BioSharing Information Resource](https://biosharing.org/)), or the [ARRIVE guidelines](http://www.plosbiology.org/article/info:doi/10.1371/journal.pbio.1000412) for reporting work involving animal research. Where applicable, authors should refer to any relevant reporting standards documents in this form.

If you have any questions, please consult our Journal Policies and/or contact us: [editorial@elifesciences.org](mailto:editorial@elifesciences.org).

**Sample-size estimation**

- You should state whether an appropriate sample size was computed when the study was being designed
- You should state the statistical method of sample size computation and any required assumptions
- If no explicit power analysis was used, you should describe how you decided what sample (replicate) size (number) to use

Please outline where this information can be found within the submission (e.g., sections or figure legends), or explain why this information doesn’t apply to your submission:

As most experiments are ensemble/bulk experiments in which phenotypes/effects arise from >1x10^6^ cells/molecules sample-size estimation were not required.

Sample sizes for statistical analysis are included in the corresponding figure legends and in the corresponding Material and Methods section at the end of the manuscript (p. 16-21) and will be shortly outlined for the experiments where shown graphs depict mean values of several biological replicates: 4A n=3, 4D n=2, 5D n=3

**Replicates**

- You should report how often each experiment was performed
- You should include a definition of biological versus technical replication
- The data obtained should be provided and sufficient information should be provided to indicate the number of independent biological and/or technical replicates
- If you encountered any outliers, you should describe how these were handled
- Criteria for exclusion/inclusion of data should be clearly stated
- High-throughput sequence data should be uploaded before submission, with a private link for reviewers provided (these are available from both GEO and ArrayExpress)

Please outline where this information can be found within the submission (e.g., sections or figure legends), or explain why this information doesn’t apply to your submission:

Information about replicates is provided in the Figure legends and in the corresponding Material and Methods section at the end of the manuscript (p. 16-21). To summarize:

- all spottings were done in 2 technical and at least 2 biological replicates (in the manuscript we always include one technical replicate of a certain experiment)

- viability experiments were conducted in replicates of 3, where for each strain and experiment colonies on 3-4 plates (containing 100-300 cells each) were counted (Fig. 4A, 5D)

- the recombination assay was performed in 2 biological replicates, where for each strain and experiment 400-600 cells were analysed (Fig. 4D)

**Statistical reporting**

- Statistical analysis methods should be described and justified
- Raw data should be presented in figures whenever informative to do so (typically when N per group is less than 10)
- For each experiment, you should identify the statistical tests used, exact values of N, definitions of center, methods of multiple test correction, and dispersion and precision measures (e.g., mean, median, SD, SEM, confidence intervals; and, for the major substantive results, a measure of effect size (e.g., Pearson's r, Cohen's d)
- Report exact p-values wherever possible alongside the summary statistics and 95% confidence intervals. These should be reported for all key questions and not only when the p-value is less than 0.05.

Please outline where this information can be found within the submission (e.g., sections or figure legends), or explain why this information doesn’t apply to your submission:

Standard Deviations have been calculated to evaluate variation between biological replicates in the survival and cross over experiments (Fig. 4A,D and 5D). In addition, p-values (applying an unpaired T-test) have been calculated using the GraphPad online tool (<https://www.graphpad.com/quickcalcs/ttest1/>) and are indicated in the corresponding experiments. The applied test and value of n can also be found in the corresponding figure legends and the corresponding Material and Methods section at the end of the manuscript (p. 16-21). Exact p-values can be found in the corresponding source data files.

(For large datasets, or papers with a very large number of statistical tests, you may upload a single table file with tests, Ns, etc., with reference to sections in the manuscript.)

**Group allocation**

- Indicate how samples were allocated into experimental groups (in the case of clinical studies, please specify allocation to treatment method); if randomization was used, please also state if restricted randomization was applied
- Indicate if masking was used during group allocation, data collection and/or data analysis

Please outline where this information can be found within the submission (e.g., sections or figure legends), or explain why this information doesn’t apply to your submission:

Not applicable

**Additional data files (“source data”)**

- We encourage you to upload relevant additional data files, such as numerical data that are represented as a graph in a figure, or as a summary table
- Where provided, these should be in the most useful format, and they can be uploaded as “Source data” files linked to a main figure or table
- Include model definition files including the full list of parameters used
- Include code used for data analysis (e.g., R, MatLab)
- Avoid stating that data files are “available upon request”

Please indicate the figures or tables for which source data files have been provided:

We have included source data files for the viability and cross over experiments that show the numerical values represented in the graphs presented in the manuscript (Fig. 4A,D and 5D)
